# Hepatic Perivascular Epithelioid Cell Tumor Mimicking Hepatocellular Carcinoma

**DOI:** 10.14309/crj.0000000000000962

**Published:** 2023-01-20

**Authors:** Rahul Harwal, Livin Jose Joseph Rosemary, Prabhakaran Raju, Sugumar Chidambaranathan, J. Bharathi Vidya Jayanthi, Naganath Babu Obla Lakshmanamoorthy

**Affiliations:** 1 Institute of Surgical Gastroenterology, Madras Medical College and Rajiv Gandhi Government General Hospital, Chennai, India

**Keywords:** liver, PEComa, perivascular epithelioid cell, HCC

## Abstract

Perivascular epithelioid cell tumors (PEComas) are rare mesenchymal neoplasms, and liver PEComas are extremely rare. They are usually discovered incidentally, and diagnostic pitfalls are frequent owing to similar imaging characteristics to other liver neoplasms. We present a patient whose evaluation was consistent with a right-sided hepatocellular carcinoma for which right hepatectomy was performed. Based on the final histopathological examination and immunohistochemistry, a diagnosis of PEComa was made. Immunohistochemistry plays a crucial role in arriving at the diagnosis, and resection represents the standard of care. A long-term follow-up is recommended because the natural history of PEComas is unpredictable.

## INTRODUCTION

Perivascular epithelioid cell tumor (PEComa) is a rare entity composed of distinctive perivascular epithelioid cells with variable melanocytic and myoid differentiation. PEComa does not have a known normal cellular counterpart, and its natural history is often unpredictable. Up to now, few cases of PEComa in various organs have been described, and treatment modalities are still controversial.

Liver PEComas are extremely rare, and patients are usually asymptomatic or diagnosed incidentally. A definite preoperative diagnosis is hard to make because of variable radiological features. Triple phase imaging in the majority of cases shows patterns of contrast enhancement mimicking hepatocellular carcinoma (HCC). Even on preoperative biopsies, an incorrect diagnosis of HCC is common owing to the epithelioid morphology of tumor cells and a trabecular pattern. Furthermore, the biological behavior of PEComa is varied. Although the majority of reported cases are benign, a minority demonstrate malignant behavior and distant metastasis.^1^ We report a case of hepatic PEComa discovered in a young woman.

## CASE REPORT

A 27-year-old woman was referred to our department with a right-sided abdominal mass and a fine needle aspiration cytology diagnosis of HCC. She was asymptomatic and had good performance (Eastern Cooperative Oncology Group 1), no weight loss, and no comorbidities.

**Figure 1.**
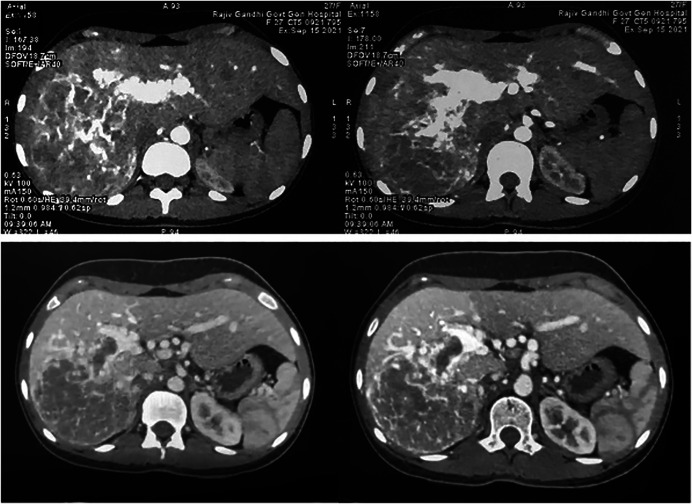
Multidetector computed tomography axial sections showing a heterogeneously enhancing mass lesion involving the right hemiliver with early arterial enhancement, persisting in subsequent phases, with prominent feeding right hepatic artery and right portal vein thrombosis.

On examination, there was a firm-to-hard nontender liver mass extending 6 cm below the right costal margin, with rounded borders. All laboratory investigations including liver function tests, viral markers, and tumor markers were normal (alpha-fetoprotein 0.4 ng/mL). Duplex ultrasound showed a 10 × 8 cm heteroechoic mass in the right liver with right portal vein invasion. Esophagogastroduodenoscopy and colonoscopy were normal. The abdominal multidetector computed tomography (Figure 1) revealed a heterogeneously enhancing mass lesion of size 10 × 10 × 13 cm involving segments V, VI, VII, and VIII of the right hemiliver with early arterial enhancement, persisting in subsequent phases, with prominent feeding right hepatic artery and right portal vein thrombosis. There were no extrahepatic lesions. The chest computed tomography was normal. Our plan was for resection for a diagnosis of right lobe HCC with right portal thrombosis.

**Figure 2.**
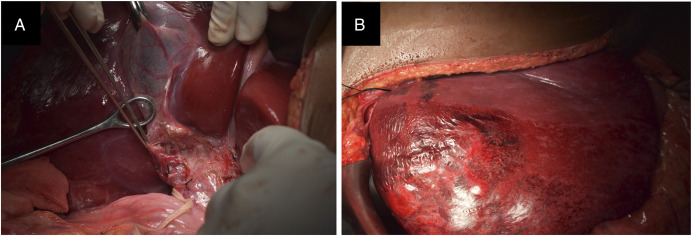
Intraoperative pictures showing (A) prominent right hepatic artery with collaterals and (B) demarcated right hemiliver after inflow control.

**Figure 3.**
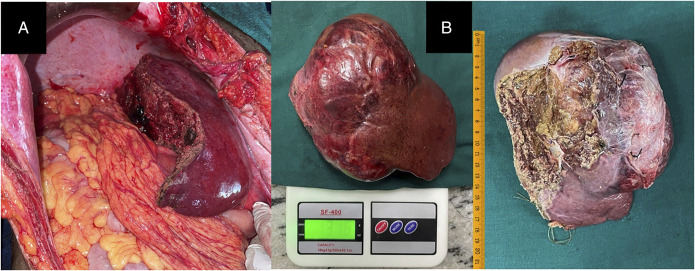
Intraoperative pictures showing (A) right hepatectomy bed and (B) specimen.

**Figure 4.**
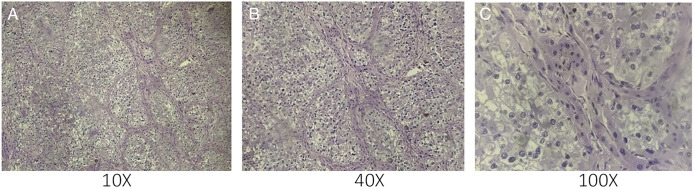
Histopathological pictures with hematoxylin-eosin stain demonstrating a circumscribed tumor with (A) sheets of polyhedral cells with eosinophilic to clear cytoplasm (B) arranged more tightly around blood vessel walls. (C) Prominent pleomorphic nuclei with dispersed chromatin, some showing conspicuous nucleoli and atypical mitotic figures.

After multidisciplinary discussion, she underwent a right hepatectomy (Figures 2 and 3). Gross examination revealed marked hypervascularity and large foci of necrosis. The postoperative course was uneventful, and she was discharged after 2 weeks. Histopathology (Figure 4) and subsequent immunohistochemistry (Table 1) were suggestive of a PEComa. Margins were free, and there was no lymphovascular/perineural invasion.

**Table 1.** Immunohistochemistry

Heat shock proteins	Scattered positivity in tumor cells
Transcription factor enhancer 3	Strong nuclear positivity in 90% tumor cells
Human melanoma black 45	Moderate positivity in 60% tumor cells
CD99	Weak to moderate staining in few scattered cells
Hepatocyte paraffin 1	Negative in tumor cells. Seen in surrounding normal hepatocytes
Glypican 3	Negative
CK7, CK20, PanCK	Negative
CD10, CD34	Negative
S100, melan A	Negative
Desmin, muscle-specific actin	Negative
Synaptophysin, CD56	Negative
CD117	Negative
Renal cell carcinoma antigen, PAX8	Negative
α-Methylacyl-coenzyme A racemase	Negative

After postoperative multidisciplinary discussion, we have kept her under long-term close observation. She is on 3-monthly follow-up (with clinical examinations, 3-monthly ultrasound and annual computed tomography). She is disease-free on the 1-year follow-up.

## DISCUSSION

PEComas are rare mesenchymal neoplasms identified mostly in middle-aged patients with a female preponderance.^1–3^ In 1992, Bonetti proposed the concept of perivascular epithelioid cells.^4^ Zamboni coined the term PEComa for the family of related neoplasms comprising AML, CCMMT, CCST, LAM, and other NOS (angiomyolipoma, clear cell myomelanocytic tumor of the falciform ligament/ligamentum teres, clear cell “sugar” lung tumor, lymphangioleiomyomatosis-like tumors, not otherwise specified).^2,5^

The histopathogenesis of perivascular epithelioid cells remains uncertain. Various hypotheses have been described regarding their origin, from undifferentiated neural crest cells,^6^ or pericytes.^5^ PEComas are also seen in the tuberous sclerosis complex.^7^

The majority of hepatic PEComas are single; however, cases with multifocal lesions have been reported.^3,8^ They pose clinical and radiological diagnostic dilemmas, mimicking metastasis or HCC, especially when multifocal. As imaging usually ends up being equivocal, histology with immunohistochemistry is essential for a definite diagnosis. In most cases, the diagnosis is confirmed only after surgery.

In 1991, human melanoma black-45 (HMB-45) positivity was reported for hepatic PEComas.^9^ Subsequent reports demonstrated both melanocytic (HMB-45 and melan-A) and smooth muscle (muscle-specific actin and desmin) markers, and other markers including S-100, CD34, and CD117.^10^ Transcription factor enhancer 3 (TFE3) positivity reflecting TFE gene rearrangements has been found in up to 20% of PEComas.^11,12^ Our patient's immunohistochemistry demonstrates predominantly melanocytic differentiation with positivity for HMB45, along with TFE3 positivity.

Folpe et al have proposed criteria for classifying the malignant potential in PEComas based on “worrisome features”: (1) tumor size >5 cm, (2) infiltrative margins, (3) high-grade nuclear atypia, (4) mitotic count >1/50 high-power fields, (5) hypercellularity, (6) vascular invasion, and (7) necrosis.^13^

It is suggested that malignant PEComas are to be considered if 2 or more of these features are present; those with none of the findings are benign, while those with size >5 cm or nuclear atypia are of uncertain malignant potential. Recent studies have shown that clinical evidence of aggressive disease is more important because the smooth muscle components in benign lesions often exhibit cytologic atypia and pleomorphism and could lead to an incorrect diagnosis of malignancy.^14^ Hypercellularity, hyperchromasia, high mitotic activity, atypical mitotic figures, and coagulative necrosis were observed in our case.

Surgical resection represents the only curative approach for primary PEComa as well as for local recurrences and metastasis because chemotherapy and radiotherapy have not demonstrated significant benefits. Only recently limited clinical studies have reported encouraging results with mTOR inhibitors in patients with metastatic PEComa.^15^

There are no established guidelines on postresection surveillance. The small proportion of PEComas that are malignant are usually aggressive tumors with early metastasis, but metastases have also been observed after 5 years. Parfitt et al documented a case of liver PEComa with benign histologic characteristics that nonetheless presented with multiple metastatic foci nearly 9 years after surgery. A long-term periodic follow-up is therefore rational.^16^ Assessment of patients and decision-making by a multidisciplinary team will result in improved diagnosis, treatment planning, and outcomes.

Liver PEComas are extremely rare and are usually diagnosed incidentally. They are usually misdiagnosed as malignant hepatic lesions preoperatively. Especially in a background of a noncirrhotic liver, primary hepatic PEComas may be one of the rare differential diagnoses. Optimal surgical resection is the best treatment option currently because results with chemotherapy or immunotherapy are yet to be validated. The long-term follow-up is necessary in all cases including benign PEComas because the natural history of the disease is not entirely known at present.

## DISCLOSURES

Author contributions: R. Harwal wrote the manuscript. J. Bharathi Vidya Jayanthi provided pathology guidance and histopathology images. LJJ Rosemary, P. Raju, S. Chidambaranathan, and NB Obla Lakshmanamoorthy revised and edited the manuscript. NB Obla Lakshmanamoorthy is the article guarantor.

Financial disclosures: None to report.

Presentation at a meeting: None.

Informed consent was obtained for this case report.
